# Structure of mitochondrial poly(A) RNA polymerase reveals the structural basis for dimerization, ATP selectivity and the SPAX4 disease phenotype

**DOI:** 10.1093/nar/gkv861

**Published:** 2015-10-10

**Authors:** Mikalai Lapkouski, B. Martin Hällberg

**Affiliations:** 1 Department of Cell and Molecular Biology, Karolinska Institutet, 171 77 Stockholm, Sweden; 2 Röntgen-Ångström-Cluster, Karolinska Institutet Outstation, Centre for Structural Systems Biology, DESY-Campus, 22607 Hamburg, Germany; 3 European Molecular Biology Laboratory, Hamburg Unit, 22607 Hamburg, Germany

## Abstract

Polyadenylation, performed by poly(A) polymerases (PAPs), is a ubiquitous post-transcriptional modification that plays key roles in multiple aspects of RNA metabolism. Although cytoplasmic and nuclear PAPs have been studied extensively, the mechanism by which mitochondrial PAP (mtPAP) selects adenosine triphosphate over other nucleotides is unknown. Furthermore, mtPAP is unique because it acts as a dimer. However, mtPAP's dimerization requirement remains enigmatic. Here, we show the structural basis for mtPAP's nucleotide selectivity, dimerization and catalysis. Our structures reveal an intricate dimerization interface that features an RNA-recognition module formed through strand complementation. Further, we propose the structural basis for the N478D mutation that drastically reduces the length of poly(A) tails on mitochondrial mRNAs in patients with spastic ataxia 4 (SPAX4), a severe and progressive neurodegenerative disease.

## INTRODUCTION

Polyadenylation is a ubiquitous mRNA post-translational modification that was first discovered in eukaryotic cells in 1960 (1). To date, most attention has been devoted to the polyadenylation that occurs in the cytosol and the nucleus, and our understanding of polyadenylation in mitochondria is, therefore, rudimentary. Mammalian mitochondrial transcription results in two large polycistronic precursors, which are processed by the RNA-processing machinery into individual tRNAs, rRNAs and mRNAs. All but one of the mRNAs are further matured by the addition of a polyadenine tail (2–4). Polyadenylation alters the fate of mRNAs in several ways. Depending on the species, it can increase mRNA stability, stimulate translation initiation, promote degradation or be required for completing certain stop codons that are not encoded in mtDNA (4–6).

In mitochondria, mRNAs are polyadenylated by mtPAP (7). Human mtPAP is active by itself *in* *vitro* and does not require protein co-factors and can use adenosine triphosphate (ATP), uridine triphosphate (UTP), cytidine triphosphate (CTP) and guanosine triphosphate (GTP) as substrates, but exhibits the highest selectivity toward ATP (8). However, the basis for this selection remains elusive. Moreover, mtPAP belongs to the non-canonical nucleotidyl transferase superfamily (9), which also includes a growing number of terminal uridylyl transferases (TUTs) involved in an array of regulatory RNA pathways. For example, the TUT activity of Cid1 promotes mRNA degradation of polyadenylated and 3′-trimmed transcripts in *Schizosaccharomyces* *pombe* (10,11). Furthermore, the mammalian RNA uridylation enzymes TUT4 and TUT7 enhance decay of microRNA. In addition, TUT4 induces rapid decay of histone mRNA, strengthening a role for oligo-U-tails as a molecular mark for mRNA degradation (12).

The structures of PAPs: bovine PAPα (13), yeast Pap1 (14), human PAPγ (15), TUTs: RET2 (16), MEAT1 (17), TUT4 (18) and Cid1 (19–21) display an enzymatic core featuring a catalytic and central domain (9). Moreover, for some non-canonical nucleotidyl transferases, the structural basis for nucleotide selectivity has also been revealed through co-crystal structures. For example, in Cid1, a single histidine (H336) in the nucleotide recognition motif is predominantly responsible for discriminating uracil over other bases, with H336A transforming its TUT activity into a PAP activity (19).

The homozygous p.N478D missense mutation in *MTPAP* causes spastic ataxia autosomal recessive Type 4 (SPAX4), a severe progressive neurodegenerative human disorder characterized by profound muscle-tone abnormalities and optic atrophy (22). The mutation diminishes mtPAP's polymerization activity, leading to an alteration in poly(A) tail lengths of representative mRNA transcripts, which dysregulates post-transcriptional expression and results in a pathogenic lack of key respiratory–chain complexes (23). This mutation does not change the oligomeric state of the protein or alter its localization (23). The molecular bases for the mutation's effect on mtPAP activity are unknown.

The crystal structure of a truncated, inactive variant of human mtPAP reveals that it is organized as a functional dimer (8). The active site, located in the cleft between the palm and fingers domains, is composed of three catalytic aspartates, including one that was mutated to assist in crystallization. However, large unresolved regions are present in the model, including the region that contains the N478 residue implicated in SPAX4. Moreover, attempts to produce nucleotide-bound crystals failed. The unsuitability of human mtPAP for structural studies, its poor *in* *vitro* activity (23), and the absence of nucleotide-bound structures motivated us to search for an ortholog that is appropriate for structural and biochemical studies. In our work, we purified an active form of chicken mtPAP and determined the crystal structures of the mature wild-type (WT) enzyme (residues 37–568) alone and in complex with ATPγS, UTP, GTP and CTP substrates to 1.82–3.1 Å resolution.

## MATERIALS AND METHODS

### Proteins and DNA

The *Gallus gallus* codon-optimized *MTPAP* gene excluding the mitochondrial-targeting sequence (according to Mitoprot (24)) (residues 37–568) was cloned into the expression construct pNIC28-BsaI using a ligation-independent cloning procedure. The protein was expressed in KRX/pRARE2 cells cultured in LB media. Expression was induced at OD_600_ = 0.6 by adding 0.5 mM IPTG and 0.1% rhamnose; induction was at 30°C for 4 h. The cell pellet was resuspended in buffer A (25 mM Tris, pH 8.5, 500 mM NaCl and 2.5% (w/v) glycerol) in the presence of a protease-inhibitor cocktail and cells were disrupted using sonication. After centrifugation at 10 000 rpm, the soluble fraction was loaded onto a 5-ml HisTrap column (GE Healthcare), washed with 10 column volumes of buffer A containing 25 and then 50 mM imidazole, and eluted using buffer A containing 250 mM imidazole. His-tag was removed overnight by using tobacco-etch virus (TEV) protease (1:50 TEV protease to mtPAP ratio). The salt concentration was lowered to 150 mM and the sample was loaded onto a 5-ml HiTrap Heparin column (GE Healthcare), and then washed and eluted with buffer A containing 500 mM NaCl. Pooled fractions were diluted to obtain a final NaCl concentration of 200 mM, concentrated to 19 mg/ml, snap frozen in liquid nitrogen and stored at −80°C. Mutants were created using the modified quick-change site-directed protocol (25). mtPAP N472D and D237N mutants were purified using the aforementioned procedure. WT and mtPAPs with residues mutated along the positively charged surface (K80–81E, K112E, K76E, R272E) were purified via one-step His-tag affinity chromatography. The MBP–mtPAP construct was created by placing the chicken mtPAP gene described above with N-terminal MBP tag into the pET3a vector using the NdeI and BamHI restriction sites. To produce an mtPAP heterodimer with only one active protomer, the D237N mtPAP kanamycin-resistant and ampicillin-resistant MBP–mtPAP WT constructs were co-expressed in KRX cells (Promega). Soluble lysate was loaded onto a 5 ml amylose resin (NEB) in buffer A, washed and eluted with buffer A containing 40 mM maltose. Next, the protein was purified on a 5-ml HisTrap column as described above for the WT.

### Crystallization, data collection and structure determination

Crystals of Apo mtPAP were grown in a hanging-drop plate (Hampton Research) by mixing 2 μl of the protein with 2 μl of the precipitant containing 0.2 M NaCl, 0.1 M Bis-Tris buffer, pH 5.5 and 20–23% (w/v) PEG 3350. The crystals were cryoprotected in the mother liquor supplemented with 20% (w/v) glycerol. Nucleotide-bound crystals of mtPAP were obtained by soaking the Apo crystals for 30 min in a solution containing 25 mM Bis-Tris, pH 5.5, 200 mM NaCl, 27% (w/v) PEG 3350 and 20% (w/v) glycerol and supplemented with 10 mM MgCl_2_ and 10 mM of nucleotide. Crystals were harvested in MicroLoops (MiTeGen) and then flash frozen in liquid nitrogen. X-ray diffraction data were collected using synchrotron radiation at different beamlines (Table 1). Datasets were indexed and processed using XDS (26) and scaled using SCALA (27). The space group was determined to be *P*2_1_2_1_2_1_ by using POINTLESS (27). The resolution cutoff used throughout was CC^1/2^ ∼0.5 (28). The Apo mtPAP structure was solved by performing molecular replacement using MOLREP (29) with the conserved polymerase core of human mtPAP as a search model (PDB code: 3PQ1). Density modification was performed in PARROT (30) and the initial model was constructed using BUCCANEER (31). Manual model building was performed in COOT (32) interspersed with refinement performed using REFMAC (33). An asymmetric unit contains two mtPAP molecules featuring a Matthews coefficient (34) calculated to be 3.32 Å^3^ Da^−1^, resulting in an estimated solvent content of 63%. Structures were comprehensively validated using PHENIX (35), which includes the MOLPROBITY suite (36). Nucleotide-bound mtPAP structures were solved by MOLREP with the refined high-resolution structure of Apo mtPAP as a search model.

**Table 1.** Summary of data collection and refinement statistics

	mtPAP–Apo	mtPAP–ATPγS	mtPAP–UTP	mtPAP–CTP	mtPAP–GTP	mtPAP(N472D)–ATPγS
Data collection						
Beamline	PXIII, SLS	ID23–1, ESRF	ID23–1, ESRF	BL-14.3, BESSY	BL-14.3, BESSY	PXIII, SLS
Space group	*P*2_1_2_1_2_1_					
Unit-cell (Å, °)	*a* = 59.39, *b* = 94.37, *c* = 191.85	*a* = 61.64, *b* = 96.70, *c* = 192.38	*a* = 59.71, *b* = 93.83, *c* = 192.44	*a* = 61.06, *b* = 95.57, *c* = 191.61	*a* = 59.75, *b* = 94.36, *c* = 192.01	*a* = 60.23, *b* = 95.22, *c* = 190.51
Resolution (Å)	40.0–1.82 (1.92–1.82)	40.0–2.50 (2.64–2.50)	40.0–2.75 (2.90–2.75)	40.0–3.10 (3.27–3.10)	40.0–2.45 (2.58–2.45)	40.0–2.75 (2.90–2.75)
Mosaicity (°)	0.1	0.15	0.24	0.17	0.38	0.30
Multiplicity	13.2 (13.0)	8.2 (8.7)	5.3 (5.4)	5.9 (6.0)	7.3 (7.6)	10.1 (10.4)
Completeness (%)	100 (100)	99.9 (100)	99.8 (100)	99.9 (100)	99.0 (98.0)	99.8 (100)
I/σ(I)	17.1 (1.3)	13.9 (1.3)	16.1 (1.9)	11.3 (1.4)	11.6 (2.1)	15.1 (1.7)
CC1/2	0.99 (0.54)	0.99 (0.50)	0.99 (0.53)	0.99 (0.52)	0.99 (0.52)	0.99 (0.57)
*R*_merge_ (%)	10.9 (204.0)	8.4 (187.3)	6.8 (90.1)	15.5 (116.2)	12.0 (92.3)	14.1 (160.8)
Refinement						
Resolution (Å)	40.0–1.82	40.0–2.50	40.0–2.75	40.0–3.10	40.0–2.45	40.0–2.75
No. reflections	92603	38577	27409	19971	38301	27694
*R*_work_ /*R*_free_	22.21/24.53	22.56/25.30	21.63/25.50	21.22/25.97	25.99/29.50	24.69/28.72
*B* factors (Å^2^)						
Protein	32.9	78.0	84.2	78.6	50.19	69.6
Mg^2+^/NTP	-/-	65.2/66.5	100.6/132.4	82.0/126.1	50.4/72.7	51.9/58.7
Water	39.2	52.0	56.5	46.6	29.2	44.9
No. atoms						
Protein	7556	7295	7436	7446	7348	7297
Water	668	25	5	4	20	5
Mg^2+^/NTP	-/-	2/73	2/58	2/58	2/45	2/62
Bond angles (°)	0.92	0.97	0.93	1.05	0.95	0.98
Bond lengths (Å)	0.005	0.005	0.005	0.006	0.005	0.005
PDB code	5a2v	5a2w	5a2y	5a2x	5a2z	5a30

The last resolution shell details are given in parentheses. The CC_1/2_ statistics and cutoffs are based on (28).

Sequence and structural alignments were performed in ClustalW (37) and rendered using ESPript (38). Protein interfaces were analyzed using the PDBe PISA web server at the European Bioinformatics Institute (39) and residue conservation was mapped onto the crystal structures using ProtSkin (40). Structural figures were prepared using PyMOL (www.pymol.org).

### *In* *vitro* polyadenylation assay

A 25-nt ssRNA was designed for activity assays with the sequence GCUACGCCGGCCUCCCCCCAAUCUA. This substrate mimics the terminal part of the chicken ND1 mRNA, where the addition of an adenine to the 3′ completes the stop codon UAA. The ssRNA was synthesized by Integrated DNA Technologies. Polyadenylation reactions were performed at 37°C for 30 min in a buffer containing 25 mM Tris, pH 8.5, 60 mM NaCl, 5 mM MgCl_2_, 0.1 mM ethylenediaminetetraacetic acid (EDTA), and 0.5 mM ATP and contained 1 μM RNA and 5, 25 and 50 nM mtPAP in a 20-μl reaction volume. Reactions were terminated by adding formamide solution that contained 10 mM EDTA and a loading dye, and samples were resolved on 14 or 20% TBE-urea denaturing gels and stained with SYBR Green II RNA gel stain (Life Technologies).

## RESULTS

### Overall structure of mtPAP

**Figure 1.**
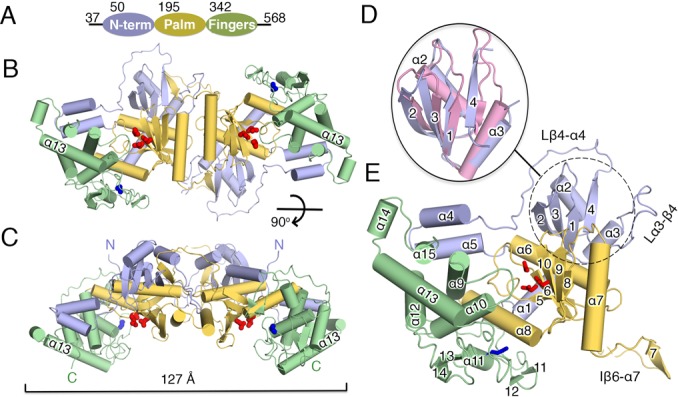
Crystal structure of mtPAP. (**A**) Diagram of mtPAP domains with their boundaries indicated by residue numbers. (**B**) Front and (**C**) top views of the mtPAP homodimer. The three mtPAP domains are colored as in (A). The dimension of the dimer is marked by a bracket. (**D**) Superposition of the NTD of mtPAP (dotted circle) and the RRM domain 4 (pink) of polypyrimidine tract-binding protein (PDB code: 4CQ1), with the secondary-structure elements of mtPAP numbered. (**E**) The mtPAP monomer with secondary-structure elements labeled. The active sites are highlighted by the three carboxylates, which are shown as red sticks. Blue sticks indicate the N472 residue implicated in SPAX4 (see main text). Loops discussed in the text are indicated.

We set out to understand the structural basis for mtPAP's substrate selectivity, its obligatory dimerization and the structural basis for the disease-causing SPAX4 mutation. Given that previous attempts to crystallize full-length WT human mtPAP have been unsuccessful (8), we tried a number of different homologs for solubility, purifiability and crystallizability. We finally selected the chicken mtPAP homolog that is the easiest to express and purify (‘Materials and Methods’ section and Supplementary Figure S1) and that is ∼60% identical to human mtPAP (Supplementary Figure S2). We crystallized mature (37–568 aa) chicken mtPAP and solved its structure to a resolution of 1.82 Å. We were able to model residues 52–527 of the protein, and only two short loop regions were poorly defined (molecule A, Mol-A: 248–253 aa; molecule B, Mol-B: 127–130 aa). The purified mtPAP crystallized as a dimer as observed in solution (Supplementary Figure S1B). mtPAP consisted of an N-terminal domain (NTD: 52–194 aa) and (using the domain nomenclature of the DNA Pol β family) of palm (195–341 aa) and fingers (342–527 aa) domains (Figure 1A–C).

The NTD's core folds into a four-stranded β-sheet, with two helices packed on one face adopting the canonical β1–α–β2–β3–α–β4 topology of an RNA-recognition motif (RRM) domain (41); the Cα RMSD was ∼1.5 Å to several RRM-containing proteins (Figure 1D and E) with no apparent sequence identity. RRM domains function in RNA recognition, protein–protein interactions (42) and as demonstrated recently, in homodimerization (43). The core of the NTD is connected to the palm domain through a 17-residue loop Lβ4–α4 (the loop between β4 and α4, 145–166 aa), which continues into a two-helix (α4 and α5) protrusion. The protrusion traverses the back of the active site and stacks against the fingers domain (Figure 1E).

The palm domain is composed of three α-helices (α6–α8) and a mixed five-stranded β-sheet core (β5–β6–β8–β9–β10) that forms one wall of the active site and harbors the catalytic triad conserved in all members of the Pol β family: D237, located at the base of β6; D239 on strand β6; and D319, which belongs to β10 (Figure 1E). A long insertion between β6 and α7 (Iβ6–α7, residues 244–270: Figure 1E) is directly involved in the dimerization interface. The fingers domain is mostly helical, with helices α9-α10 forming one wall of the active-site cleft and α12–α13 stacking behind (Figure 1E). Residues 384–430 between α10 and α12 are mostly in the coil-coiled form and contain only one helix (α11) and two pairs of short strands (β11–β12 and β13–β14), with the first pair nearly covering the active-site entry and extending toward the palm domain. The loop Lα14–β13 (462–485 aa) is well resolved and harbors N472 (human counterpart: N478), whose mutation to Asp is implicated in SPAX4 (Figure 1E).

### mtPAP dimerization

**Figure 2.**
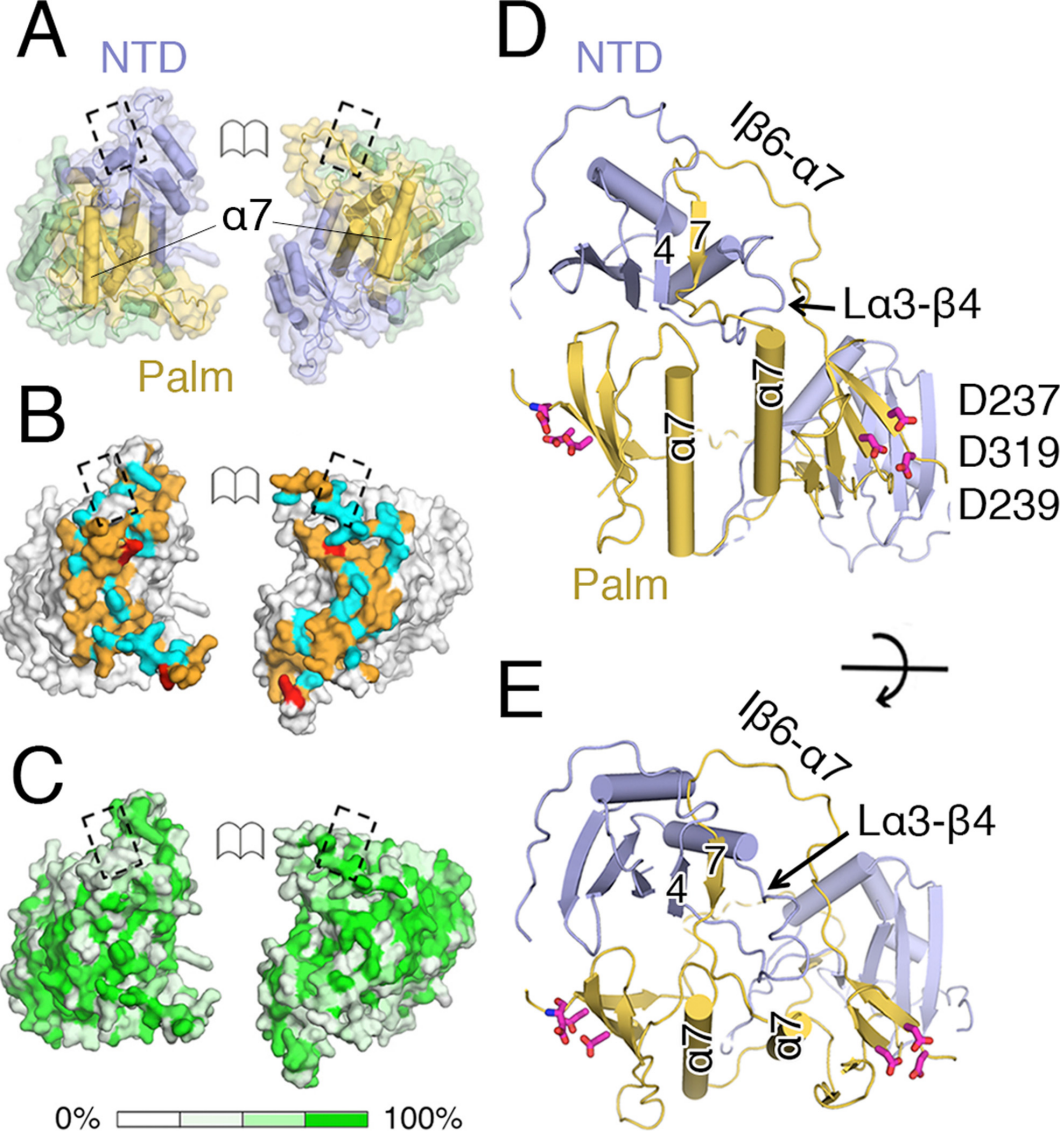
mtPAP dimer. (**A**) Open-book view of the mtPAP dimer's intermolecular interface; the domains are colored as in Figure 2 and are interacting with each other; the α7 of each monomer is shown. The dashed boxes indicate matching surfaces. (**B**) View as in (A) with the surfaces colored according to the nature of dimerization contacts. Red: salt-bridging residues; cyan: residues that form putative hydrogen bonds; orange: residues that form van der Waals contacts. (**C**) Molecular surface of mtPAP oriented as in (A), with the surface shaded according to the degree of conservation among mtPAP family members. The mtPAPs and their dimer-interface residues are typically well conserved. (**D** and **E**) Near-orthogonal views highlighting regions involved in the dimerization interface including α7, loop Lα3–β4 and insertion Iβ6–α7. Colors are as in (D): red sticks: catalytic carboxylates.

The crystal structure revealed a tight symmetric dimer featuring an extensive (7260 Å^2^) area buried upon assembly. The Cα RMSD between the two chains is 0.9 Å. The dimerization interface, indispensable for activity (8), is formed by the NTD and palm domain, mainly through hydrogen bonds and van der Waals interactions (Figure 2A and B). An extensive interface is present between two palm domains, with the α7 helices interacting with each other through a network of main-chain hydrogen bonds and van der Waals interactions (Figure 2A and B). Interestingly, the interface residues are mostly well conserved between mtPAPs (Figure 2C and Supplementary Figure S2). Loop Lα3–β4 (121–138 aa) from the NTD extends across the central axis of one dimer toward the second molecule and forms a hook, and the insertion Iβ6–α7 of one palm domain of the second molecule loops onto this hook (Figure 2D and E). Here, the Iβ6–α7 of a palm domain of one protomer in the dimer complements an additional β-strand (β7) on the far side of the four-stranded β-sheet core of the NTD next to β4 of the other protomer (Figure 2D and E).

### Comparison to the human mtPAP and fission-yeast Cid1 structures

Chicken mtPAP is structurally similar to human mtPAP (Cα RMSD 1.42 Å) (Supplementary Figure S3A). Prior to our work, the 3.1 Å structure of the human mtPAP was the only mtPAP structure available (8). There, a truncated (44–538 aa) construct of human mtPAP was crystallized as an active-site mutant D325A, which reportedly facilitated the crystallization. The refined dimeric model of human mtPAP contains poorly ordered regions that mainly belong to the NTD and regions of the fingers domain. The core secondary-structure elements of the palm and fingers domains of chicken mtPAP are structurally similar to those of human mtPAP. However, due to the higher resolution of our data, major deviations in the assignment of the NTD regions, as compared to the human model, were observed. For example, residues 255–263 of a 255–273 β hairpin of one monomer (chain B) of the human mtPAP structure in fact belong to another monomer in our high-resolution full-length structure (residues 134–142, Mol-A and β4 in Figure 1D and Supplementary Figure S3A). Additionally, only a few elements belonging to the 392–491 region of the fingers domain that form the base of the active-site cleft are resolved or built as poly-alanine models in the structure of human mtPAP (8). Therefore, a comparison of these functionally important regions is not possible.

The completeness of our mtPAP model allowed us to observe the structural similarities to the terminal uridine transferase family member—cytoplasmic poly(U) polymerase Cid1 from *S*.*pombe* (Cα RMSD 1.74 Å) (44), showing ∼20% sequence identity (Supplementary Figure S3B). The N-terminal catalytic and central domains of Cid1 superimpose well with the corresponding palm and fingers domains of mtPAP. However, there is no counterpart to the mtPAP's NTD in Cid1 and Cid1 is a monomer. Therefore, all elements involved in the dimerization interface are unique to mtPAP, including the palm-domain insertion Iβ6–α7. Comparing the active-site cleft of mtPAP with that of the closed form of Cid1 (PDB code: 4UD5) (44) revealed a further closure of the cleft in mtPAP due to a movement toward the fingers domain by the β8–β9, concomitantly with a stacking on the other side of the dimerization-mediating α7 (Supplementary Figure S3B). Additionally, a loop between α9–α10 (residues 360–369) with Ser363 on its tip of the fingers domain points to the active site (Supplementary Figure S3B).

### mtPAP–ATPγS structure

**Figure 3.**
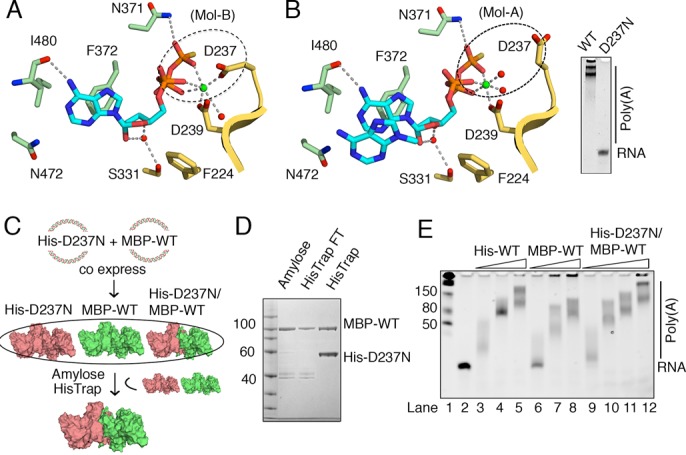
ATPγS-bound structure of mtPAP. (**A**) Coordination of ATPγS by mtPAP in Mol-B. ATPγS and coordinating residues are shown as sticks; Mg^2+^ as a green sphere; waters as red spheres; and key interactions as dotted lines. The region highlighted by the dashed oval shows that catalytic D237 is involved in Mg^2+^ coordination. D319 of the catalytic triad is not shown for clarity. (**B**) Same as (A) but in Mol-A. ATPγS and the stacking adenine ring of the second ATPγS are shown as sticks. Unlike in (A), the dashed oval highlights the absence of D237-Mg^2+^ coordination. D237 is crucial for poly(A) synthesis, as seen by the absence of the poly(A) product on the TBE-urea gel for the D237N mtPAP mutant. (**C**) Scheme for mtPAP-heterodimer production using MBP-tagged WT and His-tag D237N mtPAP constructs with different antibiotic resistance. Co-expression and purification via amylose and HisTrap affinity columns enables the selective isolation of mtPAP heterodimer where one monomer is inactive because of D237N mutation. (**D**) SDS protein gel of the mtPAP heterodimer produced by steps in (C). (**E**) Analysis of the polymerization activity of samples obtained in (C) as compared to the His-WT homodimer toward the ssRNA substrate (1 μM), 0.5 mM ATP and mtPAP concentration (5, 25 and 50 nM for His-WT and MBP-WT; 5, 25, 50 and 100 nM for MBP-WT/His-D237N); products were examined using a denaturing TBE-urea gel and stained with SYBR Green II RNA.

**Figure 4.**
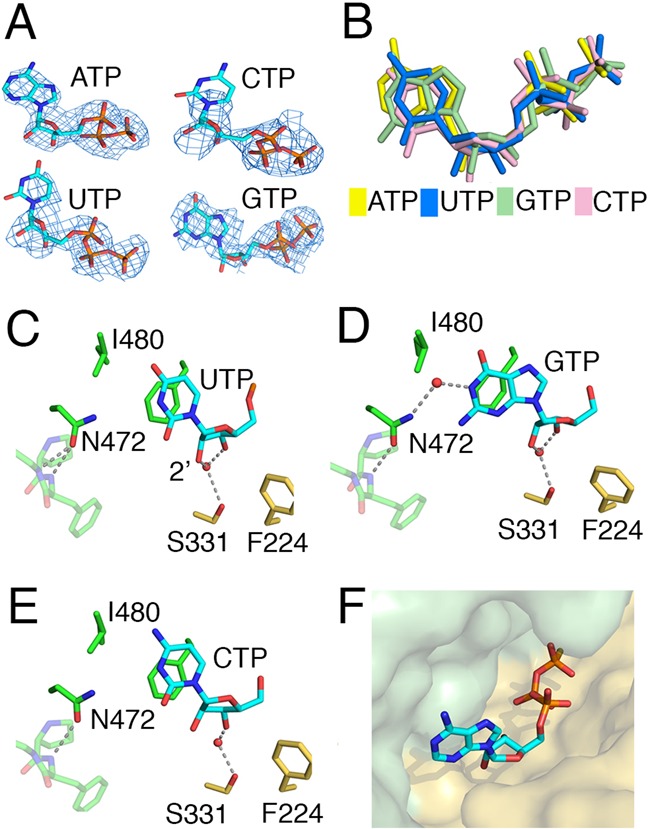
(**A**) Nucleotides in the mtPAP structures from Mol-B are shown with the simulated annealing *Fo-Fc* omit map (blue mesh), contoured at 3.0 σ for ATPγS, 2.0 σ for UTP and CTP, and 2.5 σ for GTP. (**B**) The relative positions of all four bound triphosphate ribonucleotides derived from superpositioning the mtPAP nucleotide-bound structures. (**C**–**E**) Key ribose/base interactions between mtPAP and UTP, GTP, and CTP, as found in Mol-B in the bound structures of mtPAP. The same interactions were observed in Mol-A, except in the case of the GTP where only the triphosphate moiety was modeled. (**F**) Surface representation of mtPAP nucleotide binding pocket with ATPγS shown as a stick model.

*In* *vivo*, post-transcriptional maturation of human mitochondrial mRNAs takes place in the form of 3′-tail synthesis catalyzed by mtPAP. The tails formed are composed almost exclusively of adenosine residues (7) and to gain comprehensive understanding of mtPAP's ATP specificity, we determined the crystal structure of mtPAP complexed with the non-hydrolyzable ATP analog ATPγS in the presence of Mg^2+^ (Table 1). The two hook-and-loop regions of Lα3–β4 and Iβ6–α7 that form a part of the dimerization interface (Figures 1E and 2D and E) and that are resolved on one side of the Apo dimer were disordered in the ATPγS complex structure. The ATPγS electron density was well defined (Figure 4A). ATP is primarily recognized through its triphosphate moiety by residues from the palm and fingers domains (Figure 3A). The γ-phosphate oxygen is bound through contacts to the side chain of N371. The β-phosphate oxygen group is bound by the side chain of S226 and D239 binds both the α- and β-phosphate oxygens of ATPγS (Figure 3A). The ribose moiety is sandwiched between the aromatic side chains of F372 and F224, which are highly conserved among mtPAPs (Figure 3A and Supplementary Figure S2). The 2′-hydroxyl group forms a water-mediated hydrogen bond with the side chain of S331, thereby helping the enzyme discriminate between ATP and dATP. The adenine base is in the *anti*-conformation, with F372 and L480 positioned on one side of the ring. N6 of the adenine ring donates a hydrogen bond to the main-chain carbonyl group of I480, with the interatomic distance being 3.3 Å. We observed density for a second adenine ring from ATPγS that was stacked ∼4 Å from the primary ATPγS bound in the active site. This second ring takes the position of an aligned incoming 3′ RNA base. The second ring was modeled in one chain only (Figure 3B); in the other chain, the density was too weak for the ring to be modeled.

Intriguingly, the Mg^2+^ coordination is distinct in the two protomers: in Mol-B, Mg^2+^ is coordinated by the carboxyl groups of the conserved residues D237 and D239 (Figure 3A), whereas in Mol-A, the side chain of D237 is flipped away 6 Å from the Mg^2+^ (Figure 3B). Proper coordination of Mg^2+^ by D237 is important, and D237N substitution completely eliminates the poly(A) activity (Figure 3B). To determine whether mutating one active site has an effect on the poly(A) activity of the dimer, we created a His-tagged D237N mtPAP construct with blocked ATPase activity and maltose-binding protein-tagged WT mtPAP (Figure 3C). Co-expression and two-step purification via amylose and immobilized-metal affinity chromatography columns enabled us to obtain an mtPAP heterodimer in which one monomer was active and the other was not (Figure 3C and D). The poly(A) activity of such a heterodimer was comparable to that of the WT homodimer (Figure 3E). Therefore, the two active sites can function independently of each other.

### mtPAP nucleotide selectivity

**Figure 5.**
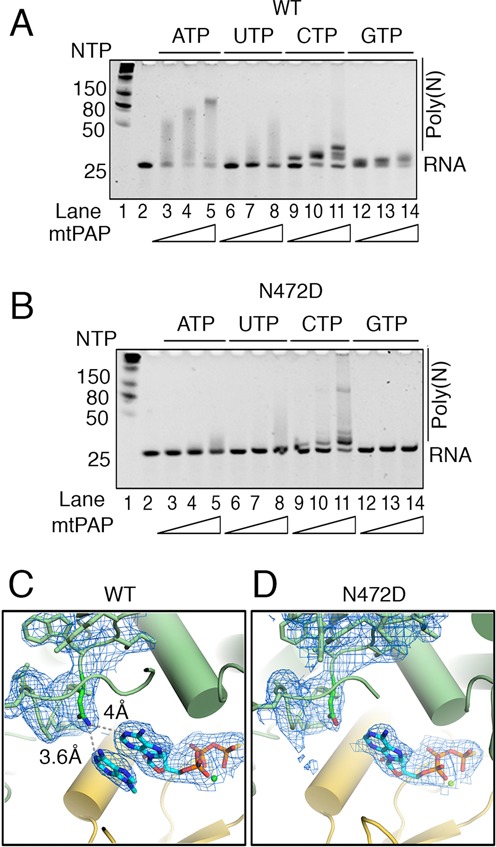
Molecular basis for the SPAX4 N472D mtPAP mutant. (**A**) Analysis of the polymerization activity of WT and (**B**) N472D mtPAP on ssRNA substrate (1 μM), shown according to nucleotides used (0.5 mM) and protein concentration (5, 25 and 50 nM); products were examined using a denaturing TBE-urea gel and SYBR Green II RNA gel staining. (**C**) Organization of the active site of the WT mtPAP Mol-A with the second adenine ring next to ATPγS molecule and the loop containing N472 (atoms are color coded) implicated in disease are shown as a stick model with corresponding 2*Fo-Fc* electron density contoured at 1 σ. Distances of ND2 to N1 atoms of both adenine rings are shown. (**D**) Similar to (C) except for the active site of the N472D mtPAP-ATPγS with 2*Fo-Fc* electron density contoured at 1 σ.

Since mtPAP can use UTP, CTP and GTP as co-substrates *in* *vitro* (Figure 5A), we determined the structures of mtPAP complexed with these nucleotides (Table 1). Unlike with the ATPγS-bound structure, the densities obtained for the bases and riboses were not clearly defined in the UTP- and CTP-bound structures in both chains (Figure 4A), and modeling them based on the resolved phosphate tails (Figure 4B) did not reveal any side-chain-specific contacts (Figure 4C and E). GTP was better resolved than the other two nucleotides (Figure 4A), but only in Mol-B, where it fits into the active site and creates a water-mediated contact between O6 and ND2 of N472 (Figure 4D). In Mol-A, the density for the guanine ring and ribose were poorly defined and unambiguous density was only observed for the triphosphate moiety.

Overall, the mtPAP nucleotide-binding site exhibits high surface complementarity for ATP (Figure 4F); and, in the four nucleotide-bound mtPAP structures, only ATPγS is unambiguously resolved. This suggests that initial nucleotide selection occurs in the absence of a template. Specifically, the stacking interactions of F372 and F224 clearly contribute to the selectivity toward ATP. Furthermore, a hydrogen bond is observed between N6 of the adenine base and the main-chain carbonyl of I480. This hydrogen bond is lacking in the mtPAP structures with the other nucleotides.

### Structural basis of the human SPAX4 phenotype

The replacement of N478 with Asp in human patients with SPAX4 leads to a drastic shortening of poly(A) tails in mitochondrial mRNA and the development of mitochondrial dysfunction with a neurodegeneration phenotype (22,23). In chicken mtPAP, the corresponding residue is N472. We determined the activity of N472D mtPAP *in* *vitro* and compared it to the WT enzyme (Figure 5A and B). In our crystallographic model, the N472 lies within ∼3.9 Å (Mol-A) to 4.2 Å (Mol-B) from the N1 atom of ATP's adenine moiety (Figure 5C). The quality of this region's electron-density map is high and the orientation of the amide group of the N472 side chain is clear and fixed (Figure 5C) for two reasons: first, OD1 accepts two hydrogen bonds from the obligate main-chain NH donors F474 and E475, and second, the other rotamer is sterically fixed in this position. In the current open, non-elongating structure, N472 does not directly interact with the adenine ring and by itself is not a determinant for ATP selectivity. However, the N472 side chain will be able to form direct and selective contact with the N1 atom of the adenine ring after the expected domain closure upon the binding of an RNA substrate.

To confirm that the reduction in poly(A) tail length in N472D mtPAP was not caused by compromised ATP binding, we solved the crystal structure of the N472D mtPAP-ATPγS complex (Table 1). The structure was virtually identical to the WT-ATPγS structure with a well-defined density for the ATPγS and no changes in the conformations of the surrounding residues (Figure 5C and D). However, the second adenine ring, which takes the presumed position of an aligned incoming 3′ RNA base in the WT structure (Figure 5C), was missing from both protomers in the N472D mtPAP–ATPγS complex (Figure 5D).

### Mapping of a putative RNA binding interface

**Figure 6.**
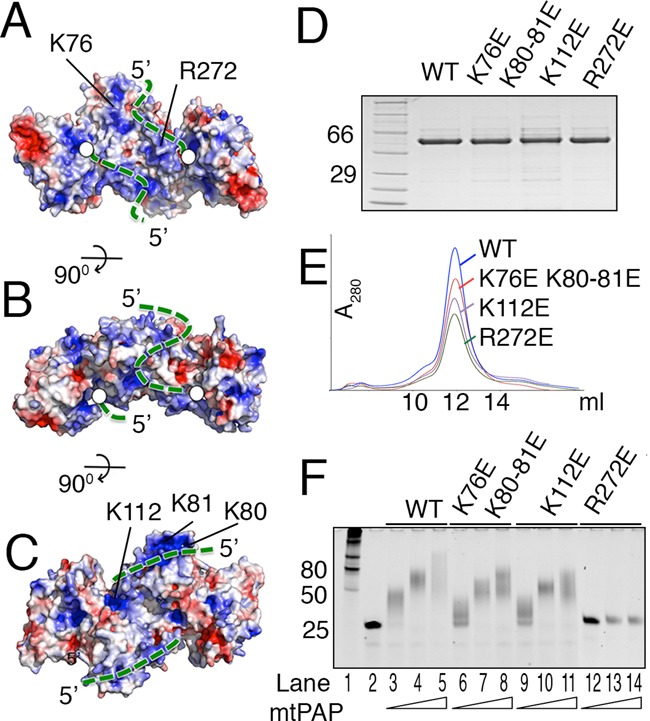
Mapping the RNA-binding path on mtPAP. (**A**) Electrostatic potential surface of the mtPAP structure. Blue and red indicate positively and negatively charged surface, respectively. The mtPAP is oriented as in Figure 2A. The probable 3′ to 5′ RNA path is indicated by the green dotted line as it follows from the active site (white circle) across the palm domain, palm-NTD dimerization interface, and along the backside of the NTD. (**B** and **C**) are orthogonal views of (A). (**D**) SDS protein gel and (**E**) Superdex-200 elution profiles of WT and mutated mtPAP proteins. (**F**) Analysis of the poly(A) polymerization activity of WT and mutated mtPAP proteins at concentrations 5, 25 and 50 nM on 25 nt ssRNA substrate (1 μM); products were examined using a denaturing TBE-urea gel and SYBR Green II RNA gel staining.

Upon dimerization, a continuous positively charged surface is formed that traverses from the active site via the palm domain (Figure 6A–C). Furthermore, this surface wraps around the NTD, and the mRNA may follow this path (Figure 6A–C). To test this, we produced mtPAP mutants with reverse-charged substitutions at several residues forming the positively charged surface (Figure 6A–D), without an effect on the stability or the dimerization state (Figure 6E). The poly(A) polymerization activity of a triple mutant K76E, K80–81E was reduced compared to the WT protein, as was also the case for the K112E mutant (Figure 6F). Most interestingly, mutation of R272E located along a probable path completely abolished poly(A) tail synthesis.

## DISCUSSION

Here, we presented the first atomic structure of WT mtPAP. Our data provide novel molecular insights into the organization, ATP selectivity and dimerization of mtPAP through its NTD. The NTD structural organization is strikingly similar to the RRMs in several RNA-binding proteins. An intriguing feature is the extension of the canonical four-stranded RNA-binding surface of NTD by an additional β-strand, supplemented by the palm domain from the other protomer in the dimer. Combined, this structure increases the surface area of NTD and may assist in mRNA binding, which displays a gain of function upon dimerization. Furthermore, a charged path is formed upon dimerization and we propose that the mRNA may follow this path (Figure 6A–C). The proposed path could be confirmed in its beginning (R272E) but mutations much farther from the active site had only a weak effect (Figure 6F). However, a larger number of positively charged residues most probably contribute to the surface formation other than those we have probed in our study and further mutations are needed to provide a cumulative effect. Thus, the positively charged surface created upon dimerization contributes to the mtPAP activity and may serve as an mRNA path. A detailed understanding of the molecular interface between mtPAP and RNA will have to await a structure of an RNA-bound complex.

The structures of mtPAP bound to each of the four ribonucleotide triphosphates reveal that the protein most efficiently accommodates ATP as judged by the defined electron density. By contrast, GTP (in one chain), UTP and CTP bases are not stabilized in the active site and are poorly resolved. We speculate that the surface complementarity of the nucleotide-binding site is one of the main factors that determine ATP selectivity. ATP is also the most abundant nucleotide in the mammalian mitochondria (45).

**Figure 7.**
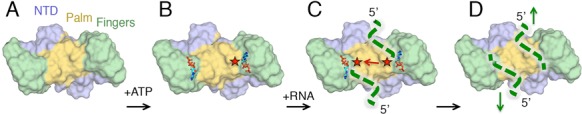
Proposed catalytic cycle of mtPAP. (**A**) Surface representation of the mtPAP with domains colored as in Figure 2A. Upon ATP binding (**B**), active-site asymmetry is created by rotation of D237. After the RNA is bound, the polymerization reaction alternates between the two active sites, (**C** and **D**), and are accompanied by the translocation events (in the direction of the green arrows).

As observed for the ATPγS-bound mtPAP structure, Mg^2+^ coordination in one monomer is optimal for ATPase reaction; whereas, in the other monomer, the side chain of the catalytic D237 is flipped away. Therefore, we propose a model in which the two monomers cannot concurrently perform polymerization and the binding of ATP makes the two active sites different (Figure 7A and B). Upon RNA binding, the polymerization reaction is likely coordinated between the two monomers with one acting at a time (Figure 7C and D). Further detailed analysis of the action of mtPAP will have to await the structure of the ternary complex.

We did not observe direct contacts between side chains and ATPγS but noted that N472 was in direct proximity to the adenine base. By itself, N472 does not determine specificity toward ATP, but its mutation to aspartate is implicated in the human SPAX4 disease phenotype characterized by drastic shortening of mRNA poly(A) tails. We propose that N472 plays a role in the precise positioning of the incoming 3′ nucleotide of mRNA in relation to the bound ATP, which is required for polymerization, and that the N472D mutation could disrupt the regular substrate-nucleotide alignment and cause a reduction in poly(A) tail synthesis. Observation of the density for the second adenine ring in our WT but not in the N472D-ATPγS structure supports the prediction that upon RNA binding and slight domain closure—N472 but not the D472 SPAX4 disease mutant—would form hydrogen bonds bridging and aligning the two adenine rings. Hence, our structure provides the molecular basis for understanding the severe progressive neurodegenerative human disorder SPAX4.

## ACCESSION NUMBERS

Atomic coordinates and structure factors have been deposited in the Protein Data Bank with accession codes 5A2V, 5A2W, 5A2X, 5A2Y, 5A2Z for Apo, ATPγS-, CTP-, UTP-, GTP-bound mtPAP, respectively, and 5A30 for the ATPγS-bound N472D mutant.

## Supplementary Material

SUPPLEMENTARY DATA
