# Supplementary material for: Structure of mitochondrial poly(A) RNA polymerase reveals the structural basis for dimerization, ATP selectivity and the SPAX4 disease phenotype
Source: Nucleic Acids Res. 2015 Oct 10;43(18):9065–75. doi: 10.1093/nar/gkv861 (PMC4605311; doi:10.1093/nar/gkv861)
Supplement: SUPPLEMENTARY DATA [file supp_43_18_9065__index.html]

Structure of mitochondrial poly(A) RNA polymerase reveals the structural basis for dimerization, ATP selectivity and the SPAX4 disease phenotype — SUPPLEMENTARY DATA 

# Structure of mitochondrial poly(A) RNA polymerase reveals the structural basis for dimerization, ATP selectivity and the SPAX4 disease phenotype

## SUPPLEMENTARY DATA

- SUPPLEMENTARY DATA
