# Supplementary material for: Structure of mitochondrial poly(A) RNA polymerase reveals the structural basis for dimerization, ATP selectivity and the SPAX4 disease phenotype
Source: Nucleic Acids Res. 2015 Oct 10;43(18):9065–75. doi: 10.1093/nar/gkv861 (PMC4605311; doi:10.1093/nar/gkv861)
Supplement: SUPPLEMENTARY DATA [file supp_gkv861_nar-01680-h-2015-File010.pdf]

Structure of mitochondrial poly(A) RNA polymerase reveals the structural basis for dimerization, ATP selectivity and the SPAX4 disease phenotype

- Supporting Information -

Mikalai Lapkouski[a,b] and B. Martin Hällberg[a,b,c,*]

[a]Department of Cell and Molecular Biology, Karolinska Institutet, 171 77 Stockholm, Sweden
[b]Röntgen-Ångström-Cluster, Karolinska Institutet Outstation, Centre for Structural Systems Biology, DESY-Campus, 22607 Hamburg, Germany
[c]European Molecular Biology Laboratory, Hamburg Unit, 22607 Hamburg, Germany

* To whom correspondence should be addressed. Tel: +46(0) 852486630; Email: Martin.Hallberg@ki.se

Supplementary Figure S1

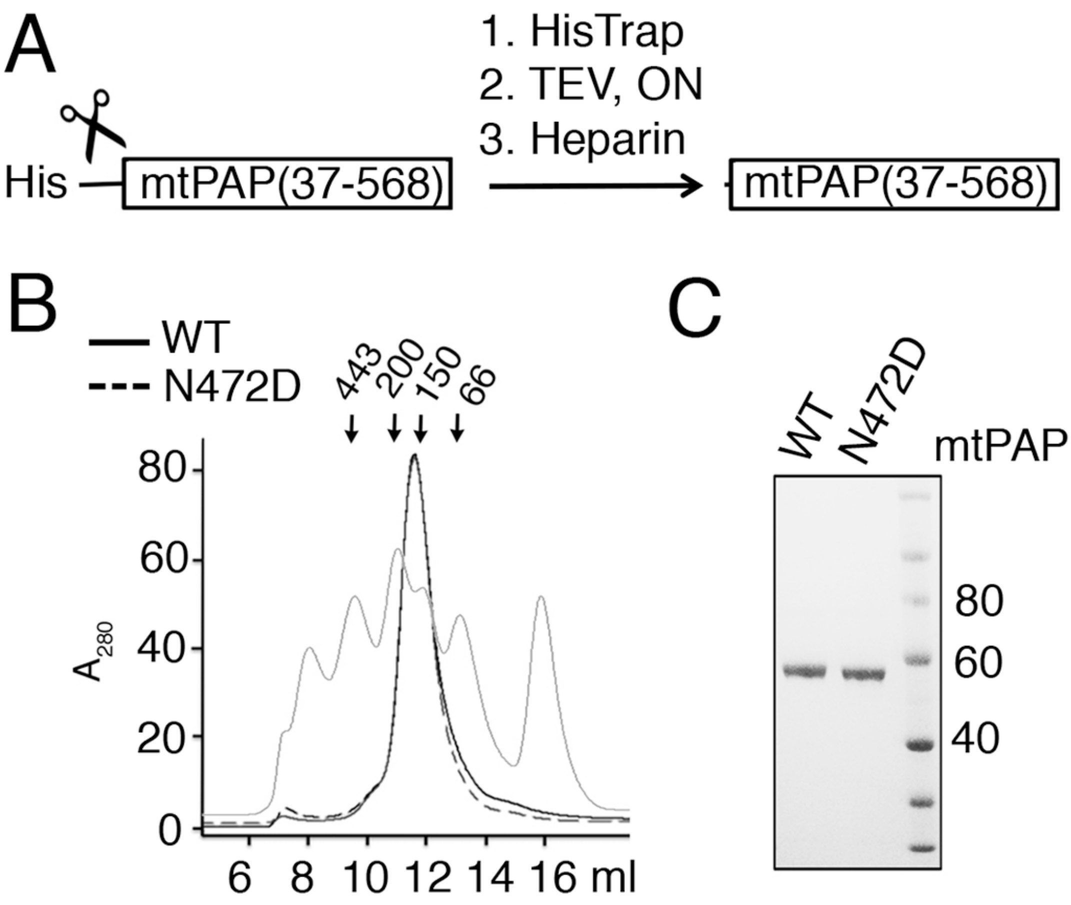

Supplementary Figure S1. Purification and activity of mtPAP proteins. **(A)** Diagram of the mtPAP construct used in this study. WT (37-568) and N472D mtPAPs were fused to a TEV protease-cleavable N-terminal His-tag and purified using a HisTrap affinity column, followed by overnight His-tag removal and a final purification over a heparin column. D372N mutant was purified the same way. **(B)** Superdex 200 10/300 gel-filtration profile of WT and N472D mutant mtPAP after purification steps shown in (A). Arrows indicate the elution points of molecular mass markers (443, 200, 150, and 66 kDa). **(C)** Protein purity shown by an SDS gel of the size-exclusion peaks from (*B*).

Supplementary Figure S2

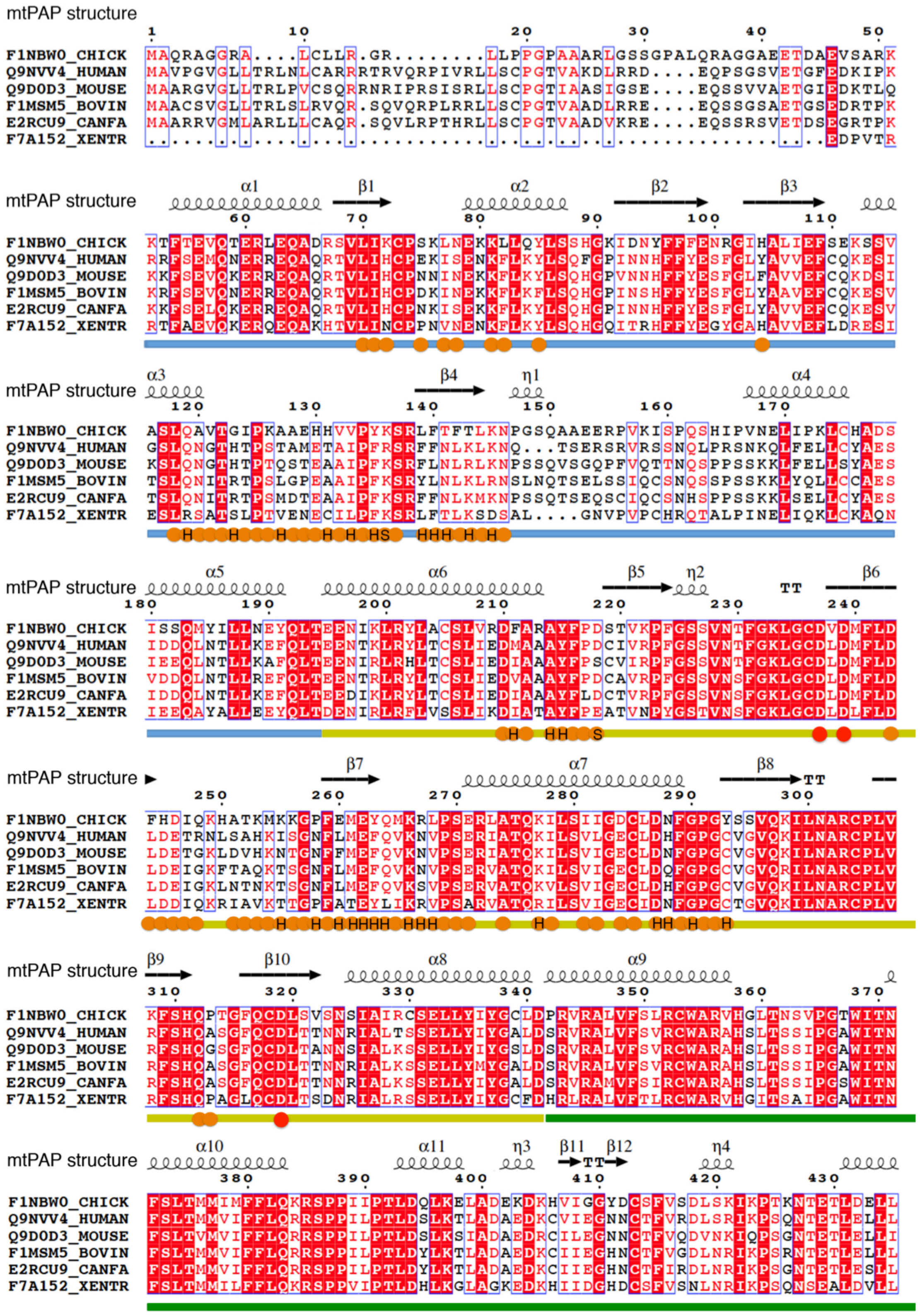

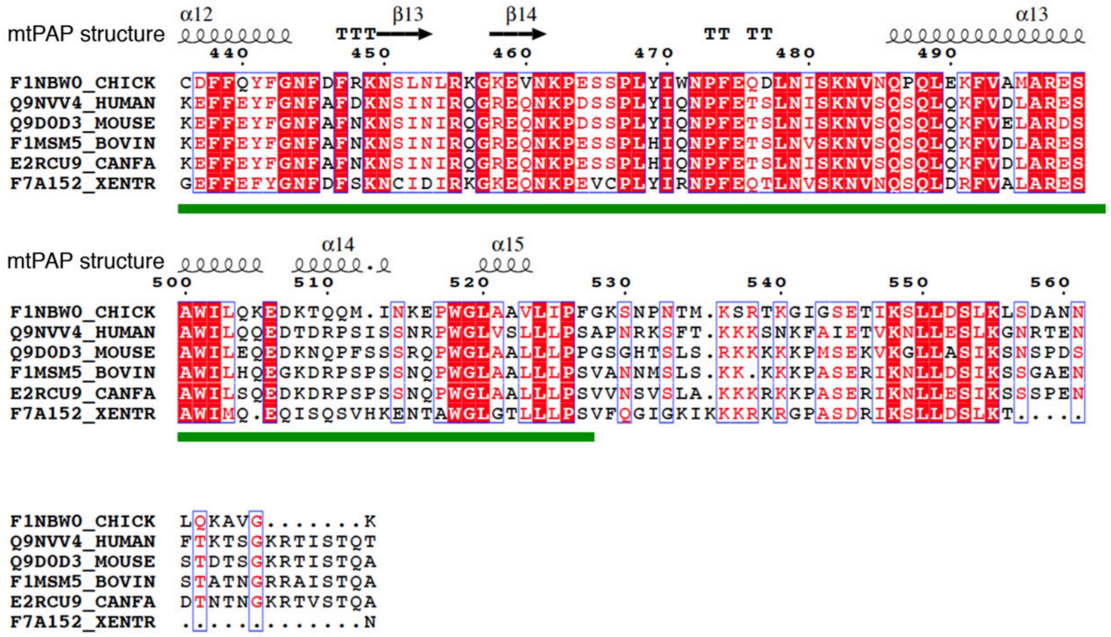

Supplementary Figure S2. Sequence alignment of mtPAP with representative family members. Residue conservation is indicated by red highlights. Secondary-structure elements are shown at the top, with assignment as in Figure 2E. Other mtPAP sequences are from the following Uniprot entries and species: F1NBW0, *Gallus gallus*; Q9NVV4, *Homo sapiens*; Q9D0D3, *Mus musculus*; F1MSM5, *Bos taurus*; E2RCU9, *Canis familiaris; and* F7A152*, Xenopus tropicalis.* Domain borders are underlined by colored lines according to Figure 2A. Catalytic aspartates are highlighted with red circles. Residues found at the dimer interface are highlighted with orange circles, with ‘H’ indicating residues involved in hydrogen bond formation and ‘S’ in a salt bridge.

Supplementary Figure S3

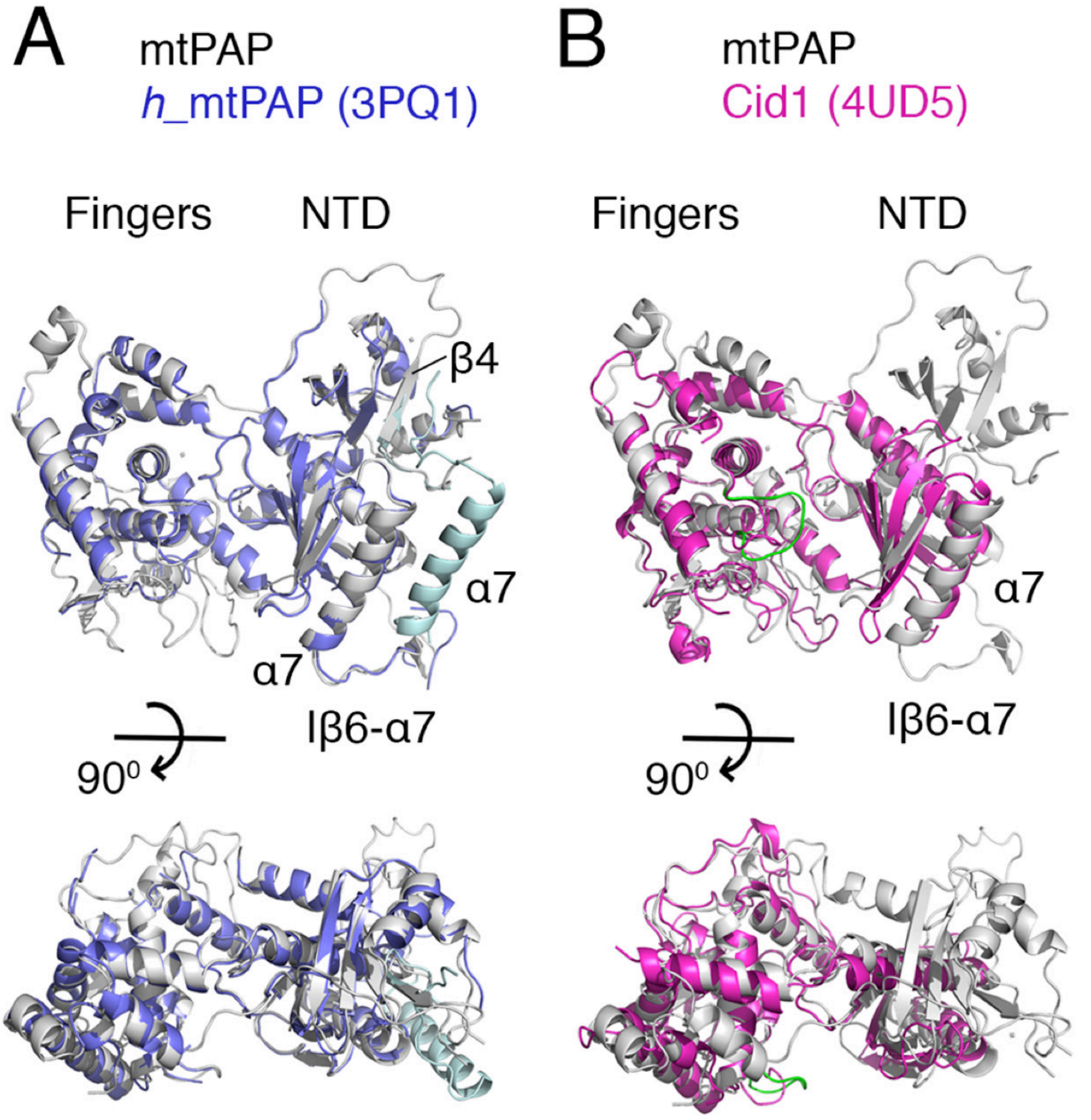

Supplementary Figure S3. Structural similarities of mtPAP. **(A)** Orthogonal view of the superposition of the domains of mtPAP (grey) and human mtPAP (blue) (PDB: 3PQ1). The α7 of the palm domain of human mtPAP for the second monomer is colored cyan. **(B)** Orthogonal view of the superposition of the domains of mtPAP (grey) and yeast Cid1 (red) (PDB: 4UD5). mtPAP domains are labeled and the palm-domain insertion unique to mtPAP is indicated. The loop between α9-α10 in mtPAP is colored green to highlight its different position from that in Cid1.
